# 

**Published:** 1878-01

**Authors:** 


### CHICAGO MEDICAL Journal and Examiner

EDITORS:

WILLIAM H. BYFORD, A.M., M.D.,
JAS. NEVINS HYDE, A.M., M.D. FERD. C. HOTZ, M.D.
E. FLETCHER INGALS, M.D.

CHICAGO MEDICAL PRESS ASSOCIATION. AUG. 2D 1875.

CHICAGO:
*PUBLISHED BY CHICAGO MEDICAL PRESS ASSOCIATION.*
188 CLARK STREET.

☞ **Read the Notice of this Journal among Advertisements.**

